# The Effects of Water Level Fluctuation on Zooplankton Communities in Shahu Lake Based on DNA Metabarcoding and Morphological Methods

**DOI:** 10.3390/ani12080950

**Published:** 2022-04-07

**Authors:** Xuemei Qiu, Quanfeng Lu, Chenchen Jia, Yuting Dai, Shan Ouyang, Xiaoping Wu

**Affiliations:** 1 School of Life Science, Nanchang University, Nanchang 330036, China; qiuxuemei2165@126.com (X.Q.); qfl89509@163.com (Q.L.); bb960301@163.com (C.J.); dyt18270925406@163.com (Y.D.); ouyangshan@ncu.edu.cn (S.O.); 2 School of Life Science, Jiangxi Science and Technology Normal University, Nanchang 330013, China

**Keywords:** China, DNA metabarcoding, morphological method, Poyang Lake, zooplankton

## Abstract

**Simple Summary:**

The water level in Shahu Lake varies greatly during an annual cycle: ~4 m deep to nearly dry. Over the course of one year we studied the relationship between zooplankton diversity and the water level in Shahu Lake using DNA metabarcoding. The morphology method was compared with the DNA metabarcoding method to see whether the results were replicable. The results were highly consistent for α-diversity and the community composition of zooplankton using both methods; both methods also showed a significant relationship between the zooplankton community composition and water level. Our research contributes to the application of the DNA metabarcoding method and aquatic ecological investigations.

**Abstract:**

Background: The water level of Poyang Lake (China) fluctuates seasonally. Shahu Lake, a smaller body of water connected to Poyang Lake during the wet season, is separated in the dry season. Due to a special fishing method termed ‘lake enclosed in autumn’, the water level is lowered and reaches its lowest point in January, which is <0.5 m deep in the middle of the lake. Our research investigated the effect of water level changes on the zooplankton community composition in Shahu Lake. Methods: We used both DNA metabarcoding method (MBC) (18S rRNA gene V4 region) and morphological method (MOI) to track the zooplankton community structure over four seasons in Shahu Lake (China). Results: Totals of 90 and 98 species of zooplankton were detected by MOI and MBC, respectively, with rotifers being the main zooplankton component. The α-diversity index of both methods increased from spring to summer and decreased from summer to autumn, reaching the lowest value in winter. NMDS and a cluster analysis showed that all zooplankton communities detected by MOI and MBC were significantly separated by season. The zooplankton community in winter was separated from that of the other three seasons, but the summer and autumn communities were more similar. Conclusions: Changes in the water level had significant effects on the zooplankton community composition. We found that MBC was more able to detect the differences in the zooplankton composition than MOI. MBC also had more advantages in copepod recognition. In our study, 37 species of copepods were detected by MBC, but only 11 species were detected by MOI. We concluded that MBC should be used to research the seasonal variations of zooplankton.

## 1. Introduction

The largest freshwater lake in China, Poyang Lake (115°55′–116°03′ E, 29°05′–29°15′ N), is located in northern Jiangxi Province. However, this lake is a floodplain–wetland complex, comprising many sub-lakes that have been formed by varied processes including hydrodynamics, sediment erosion and deposition, and artificial modification [1]. The result is a variety of depressions varying in size and shape. During floods, the sub-lakes merge with the main lake, but during periods of low water, these sub-lakes are relatively isolated from Poyang Lake. The sub-lakes have ecological values such as a large vegetation biomass, an abundant species diversity, and excellent migratory bird habitats, all of which play an important role in maintaining the wetland biodiversity and ecosystem integrity [2]. Shahu Lake is one of the main sub-lakes of the Poyang Lake National Nature Reserve. Local fishermen harvest fish from that lake using a method known as ‘lake enclosed in autumn’. In this process, a gate is opened to drain water from the sub-lake in the middle of October. Thus, the lake is basically drained until January of the next year when the gate is closed. Draining causes the surface of the lake to drop sharply to 0.3–0.4 m [1]. Due to the importance of this lake to the local fishery, we studied the spatial and temporal distribution of zooplankton in sub-lake Shahu Lake during a yearly cycle of water level changes [3].

Water level fluctuation is a key factor affecting biodiversity and ecosystem functions in aquatic habitats [4,5]. Due to the small size and short lifespan of zooplankton, the structure of their community is sensitive to environmental changes, especially in lakes with significant water level fluctuations such as flooding or water disturbance [6]. Zooplankton are an integral part of the food web, connecting phytoplankton and fish [7]; thus, they represent a major food source for fish [8] and, ultimately, the zooplankton community influences the fish density [9]. Zooplankton also are important indicators of ecological conditions [10,11] and can be used to control the standing biomass of cyanobacteria. Thus, they can contribute to ecological restoration and improved water quality [12,13]. 

As a result, monitoring zooplankton community structures provides an insight into ecosystem functions. Unfortunately, monitoring zooplankton using morphological methods to obtain qualitative and quantitative information about the zooplankton composition is both laborious and time-consuming; it requires considerable human and material support [14,15,16,17]. For example, copepods cannot be identified in their early stages of life (copepods nauplii) [15]; thus, overcoming the difficulties of identifying species and reducing the cost of classification are important problems to be solved. 

With the development of second-generation sequencing technology and the concomitant reduction of sequencing costs, DNA metabarcoding has become an important tool for biodiversity investigations. It has an efficient processing speed as well as a sensitive detection efficiency [18,19]. Studies have shown that DNA metabarcoding technology can effectively characterize the species composition of environmental DNA or a large number of biodiversity samples. As a result, this technique has become an important tool to make distinct contributions to ecological studies [20,21]. It can also be a useful tool for assessing zooplankton diversity in marine and freshwater environments [22,23,24] because DNA barcoding sequences can identify morphologically unrecognizable larval stages [25,26]. Studies have shown that the zooplankton community structure has obvious seasonal characteristics [5] with water level fluctuation being a main driving factor for zooplankton diversity [4,27]. However, there are few reports on the effects of water level fluctuations on zooplankton using DNA metabarcoding. 

We studied the impact of water level changes on the zooplankton diversity and community structure in a sub-lake (Shahu Lake) based on DNA metabarcoding technology and morphology methods. This comparative study provides theoretical support for the planning and management of water resources for biodiversity conservation and an important scientific basis for the protection and sustainable utilization of lake ecosystem biodiversity resources. 

## 2. Materials and Methods

### 2.1. Sample Collection

**Figure 1:**
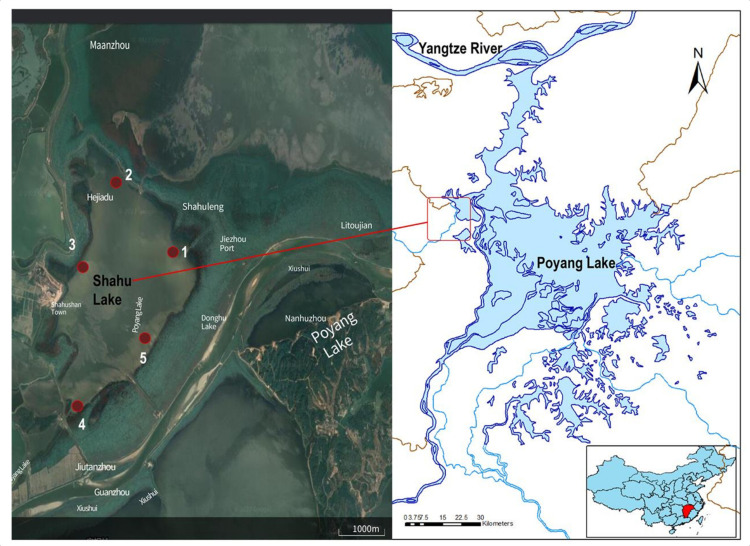
Sites sampled in the sub-lake Shahu Lake of the floodplain–wetland complex of Poyang Lake (Northern Jiangxi Province, China). The sampling sites are numbered 1, 2, 3, 4, and 5.

Zooplankton samples were collected from five sampling sites in Shahu Lake at four different times during a single hydroperiod cycle: April 2019 (spring); July 2019 (summer); October 2019 (autumn); and January 2020 (winter). For each sample, 10 L of water was filtered through a plankton net with a mesh size of 64 µm, preserving three replicates in 4% formalin for the morphological methods (MOI) [5] and two replicates in 95% ethanol for the DNA extraction (MBC) (Figure 1). 

### 2.2. Physiochemical Analysis of the Water 

At each sampling, we measured several environmental factors. A YSI 650MDS (YSI) multiparameter meter was used to measure the water temperature (°C), dissolved oxygen (mg/L), pH, salinity (mg/L), and turbidity (NTU+). The chlorophyll A concentration (mg/L) was measured by a chlorophyll meter (PCH-800); the water velocity was measured by a velocity meter (FP111, Global Water, 0.1 m/s accuracy). We used a digital sonar system (H22px handheld sonar system) to measure the water depth (m). We collected water samples for each site, preserved them in sulfuric acid (H_2_SO_4_), and refrigerated them before measuring the total nitrogen (TN, mg/L) and total phosphorus (TP, mg/L) using ultraviolet spectrophotometry. 

### 2.3. Morphological and Molecular Research Methods

We stained the morphological samples with Rose Bengal sodium salt for 24 h. Species identification and counting were conducted under an anatomical microscope (Leica, S9I) and a compound microscope (Leica, DM500). The species were identified to the species level or genus level. The larvae of the copepods could not be identified to the species level so they were all counted as one species. Zooplankton were then identified [28,29,30,31,32,33,34,35]. The method used for zooplankton counting was volume sampling based on Zhang and Huang (1995) [36].

Disposable 50 mL plastic boxes and nitrile gloves were used to collect the samples to prevent DNA contamination. In the field, plankton nets were thoroughly triple-rinsed with river water between the sample sites [37]. Each sample tube was sealed and the samples were stored at 4 °C until the DNA extraction. The DNA was extracted within two weeks. The DNA extractions and PCR amplification were conducted in a fume hood and all the disposable pipes and liquid-transferring suckers were high-temperature sterilized in advance. The DNA was extracted using a marine fish tissue DNA extraction kit (TIANamp Marine Animals DNA Kit) and performed according to the instructions of the kit. 

The PCR amplification system (25 μL) was 5 × 5 μL reaction buffer, 5 × 5 μL GC buffer, 2 μL dNTP (2.5 mM), 1 μL forward primer (10 μM), 1 μL reverse primer (10 μM), 2 μL DNA template, 8.75 μL ddH2O, and 0.25 μL Taq DNA Polymerase.

The primer sequences were as follows [38]:

Uni18S: AGGGCAAKYCTGGTGCCAGC; 

Uni18SR: GRCGGTATCTRATCGYCTT.

### 2.4. High-Throughput Sequencing and Bioinformatics

The PCR amplification products were sequenced using the Illumina MiSeq platform from Shanghai Personalbio Technology Co., Ltd. (Shanghai, China). The libraries were prepared using the TruSeq Nano DNA LT Library Prep Kit of Illumina and then the PCR amplification products were pooled to form a library for sequencing. The equimolar PCR products from each sample were used to ensure an equal contribution of each community in the final sequencing library. An Illumina MiSeq platform (San Diego, CA, USA) was used using a paired-end run of about 430 bp sequence reads after the library preparation.

Raw FASTQ files were demultiplexed and quality filtered using QIIME 1.17 and reads of a low quality (mean quality < 20; scanning window = 50; contained ambiguous ‘N’; sequence length: ≥150 bp) were discarded. UCLUST was used to cluster the operational taxonomic units (OTUs) with a 97% similarity threshold and QIIME1.17 was used to generate rarefaction curves. The Statistical Assignment Package (SAP) version 1.3.2 was used to assign the representative sequence from each OTU to a specific taxonomic group according to a reference database (the NCBI nucleotide database in GenBank).

### 2.5. Analytical Method

The dominant species were calculated by the formula below:

$$Y\;=\frac{n_{i}\times f_{i}}{N}$$

where Y is the species dominance, $n_{i}$ is the number of individuals of species *i*, N is the total number of individuals for all species, and $f_{i}$ is the frequency of occurrence of the species. When the species’ Y ≥ 0.02, it was recognized as the dominant species. For MOI, we used the density of the zooplankton for the calculation and for MBC, we used the zooplankton species and the reads.

NMDS was drawn with Primer5. SPSS 25 was used for the ANOVA analysis of the diversity index. We used R package Vegan for the ANOSIM statistics as well as for mapping and calculating the α-diversity index (the Shannon–Wiener Index, Simpson’s Diversity Index, and Pielou Evenness Index). We used R package ggvenn for the Venn diagrams. The line chart was drawn using Origin8. The image processing used Adobe Illustrator CC 2019 and the other analyses were performed using Excel 2010 for statistics and analyses.

A redundancy analysis (RDA) with 499 Monte Carlo permutations was performed using CANOCO version 4.5 to evaluate the correlation between the environmental factors and the community composition of the zooplankton. All environmental factors and the community composition of the zooplankton were log10-transformed (X + 1) to meet the assumptions of multivariate normality and to moderate the influence of extreme data [39].

## 3. Results

### 3.1. Sequence Classification Composition and Richness

**Figure 2:**
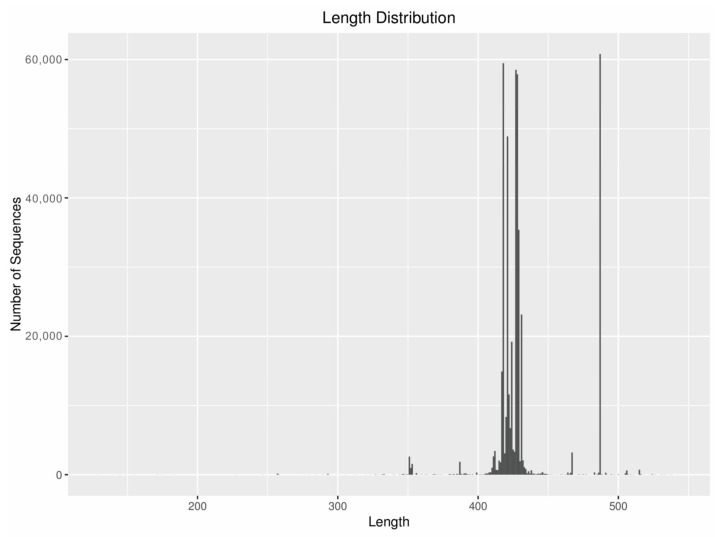
Sequence length composition diagram.

A total of 464,563 sequences and 3080 OTUs were detected by MBC. The results of the sequence alignment showed that the sequence mainly belonged to Arthropoda and rotifers; the sequence of the Arthropoda accounted for 43.43% and OTUs accounted for 33.12% whereas the sequence of the rotifers accounted for 25.90% and OTUs accounted for 16.79%. The sequence length was mostly about 428 bp (Figure 2).

### 3.2. Taxon and Species Composition

**Figure 3:**
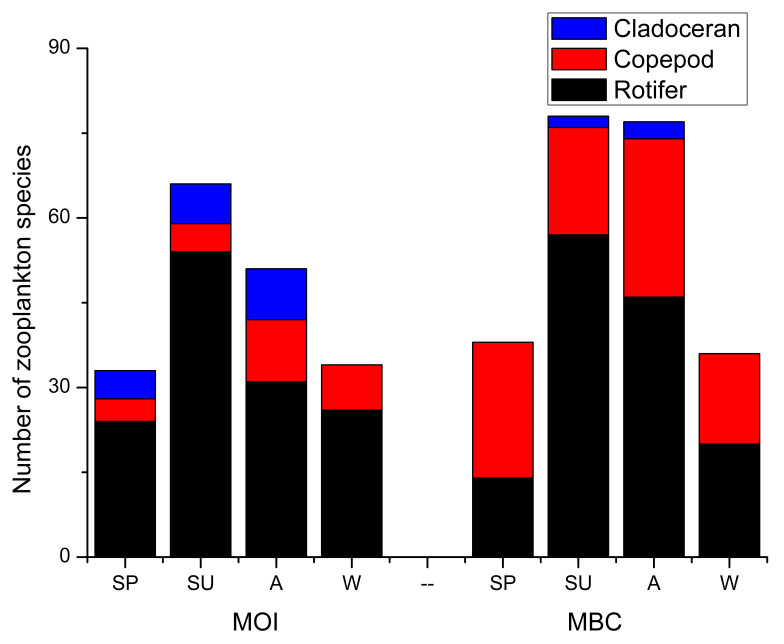
Three zooplankton species were detected by MOI and MBC. A: autumn; SP: spring; SU: summer; W: winter.

Rotifers were the main zooplankton detected by the morphological method (MOI) and DNA metabarcoding method (MBC) (Figure 3). A total of 90 species of zooplankton were detected by MOI (Table A1); they belonged to 49 genera and 22 families. A total of 98 species of zooplankton belonging to 30 families and 66 genera were detected by MBC. We detected 66 species of rotifers (26 genera) by MOI; this accounted for 73.3% of the total zooplankton species. There were 11 species of copepods (11 genera), which accounted for 12.2% of the total zooplankton species, and 13 species of cladocerans (10 genera), which accounted for 14.4% of the total zooplankton species. The results of MBC showed that 58 rotifers (39 genera) accounted for 59.2% of the total zooplankton species, 37 copepods (23 genera) accounted for 37.8% of the total zooplankton species, and 3 cladocerans belonging to 3 genera accounted for 3.1% of the total zooplankton species. 

**Figure 4:**
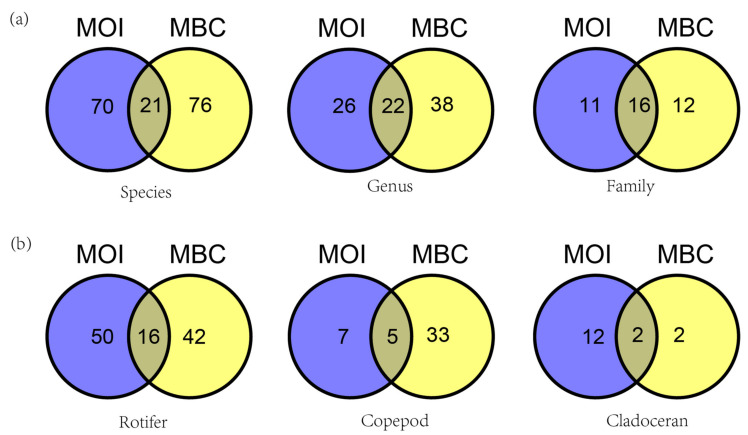
Venn diagram comparing the assessment of species composition in Shahu Lake (China). (**a**) Venn diagrams of zooplankton composition at the species, genus, and family level by MOI and MBC. (**b**) Venn diagrams of species composition of Rotifer, Copepod, and Cladoceran by MOI and MBC.

There were 21 species (12.6%), 22 genera (25.6%), and 16 families (41%) detected by both methods (MOI, MBC). As observed from the various monitored zooplankton groups, the total number of rotifer species detected by MOI and MBC was 108 and 16 were species detected by both methods, accounting for 14.8%. For copepods, a total of 45 species were detected including 5 species in both methods, accounting for 11.1%. A total of 16 cladocerans and 2 species were detected in both methods, accounting for 12.5% (Figure 4).

**Figure 5:**
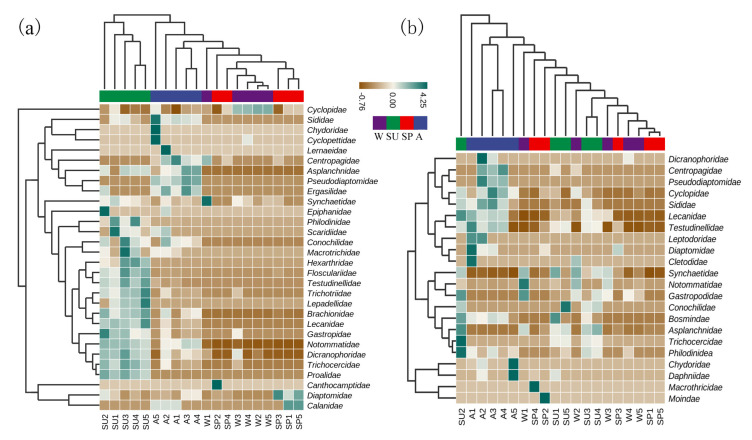
Cluster heat maps of family by MOI and MBC. (**a**) MBC; (**b**) MOI. A: autumn; SP: spring; SU: summer; W: winter. Numbers 1, 2, 3, 4, and 5 are the sample sites.

The cluster heat map showed that summer and autumn and spring and winter were clearly separated by the MBC analysis. A greater number of taxa were identified in summer and autumn and fewer in winter and spring. In contrast, MOI identified more species in summer and autumn, but only autumn had a distinct community from the rest of the seasons (Figure 5).

### 3.3. The α−Diversity Index

**Figure 6:**
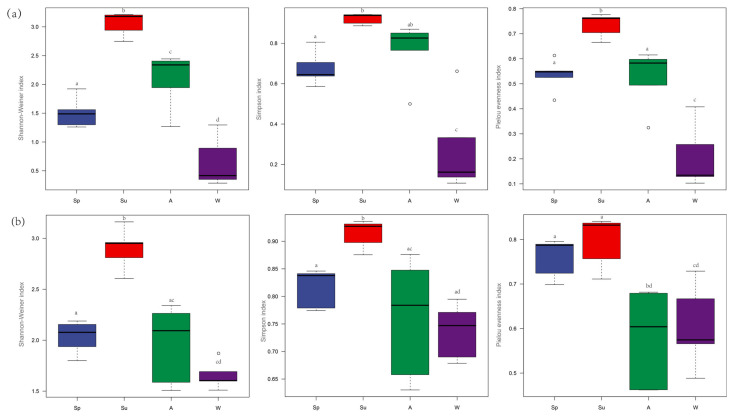
Seasonal variation of zooplankton α−diversity. (**a**) MBC; (**b**) MOI, including the Shannon–Weiner Index, Simpson’s Index, and Pielou Evenness Index. SP: spring; SU: summer; A: autumn; W: winter. a, b, c, d are marked letter, the difference is insignificant if there is a same marked letter, otherwise significant if there is a different marked letter. ο is the outliers.

The variation trend in the zooplankton α−diversity index of MOI and MBC was consistent. The α−diversity index increased from spring to summer and was significantly higher in summer than in the other seasons; the diversity index decreased from summer to autumn and reached the lowest in winter (Figure 6).

For the MOI, the Shannon–Wiener Index was significantly different between spring and summer (ANOVA, *p* = 0.000), spring and winter (ANOVA, *p* = 0.026), summer and autumn (ANOVA, *p* = 0.000), and summer and winter (ANOVA, *p* = 0.000). The Simpson Index for summer was significantly different from the other three seasons (ANOVA, *p* = 0.029, *p* = 0.002, and *p* = 0.001). Additionally, the Pielou Evenness Index was significantly different in spring and autumn (ANOVA, *p* = 0.003), spring and winter (ANOVA, *p* = 0.008), summer and winter (ANOVA, *p* = 0.002), and summer and autumn (ANOVA, *p* = 0.001) whereas the Shannon–Wiener Index, the Simpson Index, and the Pielou Evenness Index showed no difference among all the sampling points detected by MOI (ANOVA, *p* > 0.05).

For the MBC, the Shannon–Wiener Index was significantly different between spring and summer (ANOVA, *p* = 0.000), spring and autumn (ANOVA, *p* = 0.026), spring and winter (ANOVA, *p* = 0.002), summer and autumn (ANOVA, *p* = 0.001), summer and winter (ANOVA, *p* = 0.000), and autumn and winter (ANOVA, *p* = 0.000). The Simpson Index was significantly different between spring and summer (ANOVA, *p* = 0.016), spring and winter (ANOVA, *p* = 0.001), summer and winter (ANOVA, *p* = 0.000), and autumn and winter (ANOVA, *p* = 0.000). The Pielou Evenness Index was significantly different in spring and summer (ANOVA, *p* = 0.005), spring and winter (ANOVA, *p* = 0.000), summer and autumn (ANOVA, *p* = 0.003), summer and winter (ANOVA, *p* = 0.000), and autumn and winter (ANOVA, *p* = 0.000). As with MOI, the Shannon–Wiener Index, the Simpson Index, and the Pielou Evenness Index showed no difference among all the sampling points detected by MBC (ANOVA, *p* > 0.05).

### 3.4. Community Feature

**Figure 7:**
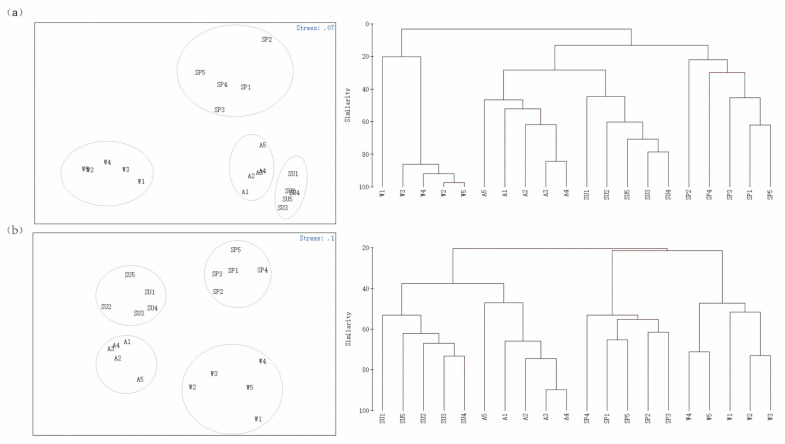
NMDS sorting diagram based on multidegree data and a similarity clustering analysis diagram. (**a**) MBC; (**b**) MOI. Sampling sites 1–5. SP: spring; SU: summer; A: autumn; W: winter.

The NMDS analysis showed that all the zooplankton communities detected by MOI and MBC were significantly separated by season (Figure 7). The cluster analysis showed that the zooplankton community in winter was separate from that of the other three seasons; the summer and autumn communities were more similar and gathered into one branch. The ANOSIM results showed that the R values of both MOI and MBC were greater than 0, indicating that the seasonal differences of the zooplankton in Shahu Lake were greater than the intra−seasonal differences. The *p*−values of both methods were 0.001, indicating that there were extremely significant differences.

**Figure 8:**
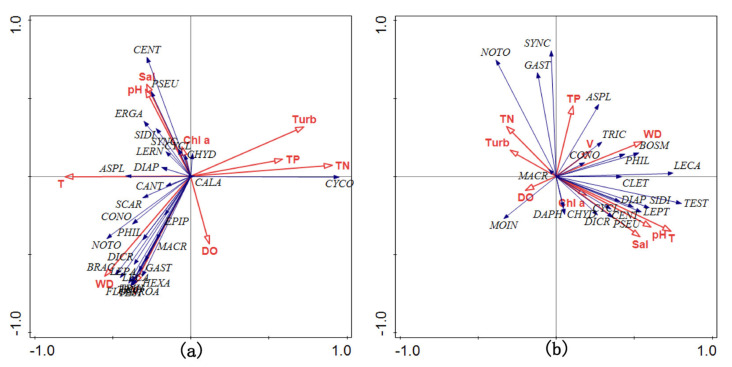
RDA of zooplankton family composition with environmental factors. (**a**) MBC; (**b**) MOI.

The RDA results (Figure 8, Table A2, Table A3) showed that the cumulative percentage difference between the zooplankton and the environment was 53.7% (MOI) and 73.1% (MBC). MOI showed that the main environmental factors affecting the zooplankton community structure were temperature (T), pH, water depth (WD), and salinity (Sal) whereas MBC showed the main environmental factors were total nitrogen (TN), water depth (WD), velocity (V), pH, and salinity (Sal).

## 4. Discussion

Changes in the water levels led to significant seasonal changes in the zooplankton community (Figure 3). There were 36 (spring), 71 (summer), 63 (autumn), and 35 (winter) species of zooplankton on average detected by MOI and MBC. Rotifer had 19 (spring), 56 (summer), 39 (autumn), and 23 (winter) among them. The number of rotifers increased at first and then decreased from spring to winter. When the water level was higher in summer and autumn, there were more rotifers. The average number of rotifer species detected by MOI and MBC was 47 in summer and autumn; the number of rotifer species then decreased in spring and winter due to low water levels. The average number of rotifer species detected by the two methods was 21 in spring and winter; the number of rotifer species decreased by 55.3% from the wet season to the dry season. The result was consistent with the research of Novotny who, by analyzing the diversity of trophic niches, found that the smaller sized rotifer had population peaks during summer [40]. Other research using morphological methods has also reported similar results [41,42,43].

We posited that variations in the lake water level caused a significant seasonal change in the zooplankton diversity [44] (Figure 6). The Shannon–Weiner Index, the Simpson Index, and the evenness index of the zooplankton showed a trend of first increasing and then decreasing from spring to winter in both MOI and MBC. The index value was the highest in summer and lowest in winter. The zooplankton diversity was highest when the water level was high and lowest when the water level was low.

Changes in the water levels caused the dominant species to change with the seasons (Table 1). Copepods were predominant in spring, autumn, and winter; rotifers were predominant in summer and the dominant rotifers were fewer in spring and winter due to low water levels. MOI showed the dominant species of rotifers totaled 5 in spring, 13 in summer, 4 in autumn, and 3 in winter. The dominant copepods in the four seasons were nauplii; *Microcyclops varicans* was dominant in spring and *Mesocyclops leuckarti* was dominant in autumn. MBC showed no dominant rotifers in spring, but 10 in summer, 5 in autumn, and 1 in winter. There were 6 dominant species of copepods in spring, 3 in summer, 4 in autumn, and 2 in winter. No dominant cladocerans were recognized by either MOI or MBC. Studies have shown that zooplankton communities are strongly influenced by climate warming and nutrient load; therefore, eutrophication and climate warming can change the zooplankton community structure and increase the dominance of small crustaceans [43]. In this study, we found that the low density of cladocerans in Shahu Lake could be related to the high degree of eutrophication in the sub−lake.

A variation in the water levels of Poyang Lake drives environmental heterogeneity, with the main abiotic factors being the water level, water temperature, electrical conductivity, total nitrogen, nitrites, and total phosphorus [45,46]. Water level fluctuations are a key factor affecting aquatic biodiversity in seasonally submerged freshwater ecosystems including in floodplain wetlands; the biomass and individual size of zooplankton are the lowest in seasonally submerged floodplain habitats [4,47]. A few studies have shown that the α−diversity of zooplankton in floodplains changes with the water lever [27,46]. Our study found that when Shahu Lake was connected to the main lake in summer, its α-diversity also reached the highest value. The water level of Shahu Lake was relatively high in summer and autumn and its species diversity was higher in these seasons than in spring and winter (Figure 6). The NMDS and cluster analysis showed that the zooplankton communities clustered together in summer and autumn (Figure 7); in spring and winter, the zooplankton communities gathered separately, also indicating that the zooplankton community was correlated with the water level. The reason may relate to the change of the phytoplankton. Using DNA metabarcoding to study trophic interactions, Zamora−Terol [48] found that the spring phytoplankton bloom, a dominance of diatom and dinoflagellate trophics with links to copepods, and summer zooplankton showed a more diverse diet dominated by cyanobacteria and heterotrophic prey. A five-year study of the phytoplankton succession in Poyang Lake by Qian et al. (2021) found that water level fluctuations greatly influenced phytoplankton succession and hydrology fluctuations had an indirect impact on a decrease of the Cyanophyta biomass. Several other studies have also showed the positive correlation between zooplankton abundance and phytoplankton biomass [49,50,51]. Although a change in the water levels did not stop the growth of zooplankton, it changed the community structure of the zooplankton; copepods were dominant in spring, autumn, and winter and rotifers were dominant in summer.

## 5. Conclusions

The effective identification of freshwater zooplankton to the species level is an important step toward understanding the structural richness and diversity of zooplankton populations [18,52,53,54]. MOI is a classic method that has been used for some time, but MBC is rapidly becoming a valuable research method. Previous studies have shown that MOI and MBC provide complementary information in biological surveys. When properly implemented, these two methods can be reliable, efficient, and low-cost in assessing the environmental impact of the marine industry [55,56]. Many studies have shown that MBC data provide the ability to identify correlations between the community structure and environmental parameters comparable with, or superior to, MOI [57]. In our study, we also found a consistency between MBC and MOI when studying the zooplankton diversity and community structure. A few researchers argue that MBC could provide wider coverage and a better resolution of taxa when compared with MOI; that outcome would strengthen biological investigations of freshwater plankton communities [58,59]. Nevertheless, generating accurate species lists from MBC data is challenging [60]. Thus, the availability of reference sequences linked to known species needs to be developed [17,61]. Several researchers have found that MBC was superior to MOI in single-sample comparisons [62,63]. In our study, the number of zooplankton species detected by MBC was higher than MOI (Table S1), but MBC was highly consistent with MOI in the study of α-diversity. However, there were also inconsistencies between MBC and MOI. For example, the species list determined by MBC was different from that of MOI. We found that three dominant species were detected by both methods, but, at the genus level, there were five dominant rotifer genera detected by both methods. This accounted for 50% of the total. The differences between the results obtained by MOI or MBC may partly be attributed to an erroneous taxonomic assignment and/or cryptic species as well as species that are hard to detect by visual means [17]. This ignores the possibility of errors in the reference database (the NCBI nucleotide database in GenBank). Nevertheless, that database is improving and recent analyses have shown that metazoan identifications in GenBank are accurate with an error rate probably < 1% at the genus level and can, therefore, be used reliably [64]. These issues will, no doubt, be improved by subsequent analyses. It seems appropriate that researchers should combine MBC with MOI at this stage, but increase the research on MBC, especially the corresponding relationship between the reads and OTU numbers generated by MBC and the density and biomass of MOI. We believe that the MOI and MBC complement each other and provide a more accurate and efficient means of freshwater ecosystem biodiversity assessments when used in combination. 

## Figures and Tables

**Table 1:** Seasonal variation of dominant species found in Shahu Lake (China) in which morphological method (MOI) and DNA metabarcoding method (MBC) were used.

Group	Species	SP	SU	A	W
Rotifers	*Anuraeopsis fissa* (MOI)	−	0.04	−	0.12
	*Ascomorpha ovalis* (MBC)	−	0.03	−	−
	*Asplanchna priodonta* (MOI)	−	0.02	−	−
	*Asplanchnopus dahlgreni* (MBC)	−	−	0.04	−
	*Asplanchna brightwellii* (MBC)	−	0.04	0.04	−
	*Brachionus angularis* (MOI)	0.06	−	−	−
	*Brachionus calyciflorus* (MOI)/(MBC)	0.14/−	−/0.14	−/0.02	−/−
	*Brachionus budapestiensis* (MOI)	−	0.03	−	−
	*Brachionus falcatus* (MOI)	−	0.04	−	−
	*Brachionus diversicornis* (MOI)	−	0.03	0.08	−
	*Brachionus urceolaris* (MBC)	−	0.07	0.04	−
	*Brachionus* sp. ^1^ (MBC)	−	0.02	−	−
	*Cephalodella gibba* (MOI)	−	−	−	0.22
	*Collotheca tenuilobata* (MBC)	−	0.03	−	−
	*Filinia longiseta* (MOI)	0.04	−	0.04	−
	*Hexarthra intermedia* (MBC)	−	0.06	−	−
	*Keratella valga* (MOI)	−	0.09	0.11	−
	*Keratella cochlearis* (MOI)	−	0.15	0.24	−
	*Keratella quadrata* (MBC)	−	0.11	0.05	−
	*Lecane* sp. ^1^ (MOI)	0.02	−	−	−
	*Polyarthra dolichoptera* (MOI)/(MBC)	0.15/−	0.06/0.03	−/−	−/−
	*Polyarthra vulgaris* (MOI)	−	0.07	−	−
	*Polyarthra remata* (MBC)	−	0.02	−	−
	*Ptygura libera* (MBC)	−	0.04	−	−
	*Rotaria neptunia* (MOI)	−	0.02	−	−
	*Synchaeta tremula* (MOI)/(MBC)	−/−	−/−	−/−	0.22/0.11
	*Trichocerca cylindrica* (MOI)	−	0.02	−	−
	*Trichocerca capucina* (MOI)	−	0.05	−	−
	*Trichocerca lophoessa* (MOI)	−	0.04	−	−
Copepods	Copepod nauplii (MOI)	0.32	0.08	0.33	0.32
	*Eucyclops serrulatus* (MBC)	0.02	−	−	−
	*Eucyclops dumonti* (MBC)	0.06	−	−	−
	*Mesocyclops leuckarti* (MOI)	−	−	0.03	−
	*Microcyclops varicans* (MOI)	0.04	−	−	−
	*Mesocyclops pehpeiensis* (MBC)	0.02	0.03	−	−
	*Mesocyclops dissimilis* (MBC)	0.3	0.05	0.06	−
	*Neodiaptomus schmackeri* (MBC)	0.31	−	−	−
	*Pseudodiaptomus inopinus* (MBC)	−	−	0.2	−
	*Sinocalanus sinensis* (MBC)	0.08	−	0.31	0.08
	*Thermocyclops* sp. ^1^ (MBC)	−	−	−	0.74
	*Thermocyclops crassus* (MBC)	−	0.05	0.12	−
	*Thermocyclops decipiens* (MBC)	−	−	−	0.03

^1^ SP, SU, A, and W are spring, summer, autumn, and winter, respectively. −: the species was not dominant in the season. MOI: the species was the dominant species in MOI. MBC: the species was the dominant species in MBC. MOI/MBC: the species was the dominant species in both MOI and MBC.

## Data Availability

All data included in this study are available upon request by contact with the corresponding author.

## Supplementary Materials

The following supporting information can be downloaded at: https://www.mdpi.com/article/10.3390/ani12080950/s1, Table S1: The mean density of zooplankton detected by MOI, and the reads of zooplankton detected by MBC.

## Appendix A

**Table A1:** The species list of MBC and MOI.

MBC				MOI			
Group	Family	Genus	Species	Group	Family	Genus	Species
Rotifers	-	*-*	Bdelloidea sp. ^1^	Rotifers	Asplanchnidae	*Asplanchna*	*Asplanchna brightwellii*
	Asplanchnidae	*Asplanchna*	*Asplanchna brightwellii*			*Asplanchna*	*Asplanchna girodi*
		*Asplanchnopus*	*Asplanchnopus dahlgreni*			*Asplanchna*	*Asplanchna priodonta*
	Brachionidae	*Brachionus*	*Brachionus calyciflorus*			*Asplanchnopus*	*Asplanchnopus multiceps*
			*Brachionus plicatilis*		Brachionidae	*Anuraeopsis*	*Anuraeopsis fissa*
			*Brachionus* sp. ^1^			*Brachionus*	*Brachionus angularis*
			*Brachionus urceolaris*			*Brachionus*	*Brachionus budapestinensis*
		*Epiphanes*	*Epiphanes senta*			*Brachionus*	*Brachionus calyciflorus*
		*Euchlanis*	*Euchlanis dilatata*			*Brachionus*	*Brachionus diversicornis*
		*Keratella*	*Keratella quadrata*			*Brachionus*	*Brachionus falcatus*
		*Lepadella*	*Lepadella rhomboides*			*Brachionus*	*Brachionus forficula*
		*Mytilina*	*Mytilina mucronata*			*Brachionus*	*Brachionus leydigii*
		*Plationus*	*Plationus patulus*			*Brachionus*	*Brachionus quadridentatus*
	Collothecidae	*Collotheca*	*Collotheca campanulata*			*Brachionus*	*Brachionus urceus*
			*Collotheca tenuilobata*			*Epiphanes*	*Epiphanes senta*
	Conochilidae	*Conochilus*	*Conochilus coenobasis*			*Euchlanis*	*Euchlanis dilatata*
			*Conochilus hippocrepis*			*Keratella*	*Keratella cochlearis*
			*Conochilus unicornis*			*Keratella*	*Keratella quadrata*
	Dicranophoridae	*Dicranophorus*	*Dicranophorus forcipatus*			*Keratella*	*Keratella valga*
		*Encentrum*	*Encentrum astridae*			*Lepadella*	*Lepadella patella*
	Flosculariidae	*Floscularia*	*Floscularia armata*			*Notholca*	*Notholca labis*
		*Lacinularia*	*Lacinularia flosculosa*			*Plationus*	*Plationus patulus*
		*Limnias*	*Limnias ceratophylli*		Conochilidae	*Conochilus*	*Conochilus unicornis*
			*Limnias melicerta*		Dicranophoridae	*Dicranophorus*	*Dicranophorus forcipatus*
		*Pentatrocha*	*Pentatrocha gigantea*		Dicranophoridae	*Dicranophorus*	*Dicranophorus luetkeni*
		*Ptygura*	*Ptygura libera*		Gastropodidae	*Ascomorpha*	*Ascomorpha ecaudis*
		*Sinantherina*	*Sinantherina ariprepes*			*Ascomorpha*	*Ascomorpha ovalis*
			*Sinantherina semibullata*			*Ascomorpha*	*Ascomorpha saltans*
			*Sinantherina socialis*			*Gastropus*	*Gastropus hyptopus*
	Gastropodidae	*Ascomorpha*	*Ascomorpha ovalis*		Lecanidae	*Lecane*	*Lecane bulla*
	Lecanidae	*Lecane*	*Lecane bulla*			*Lecane*	*Lecane cornuta*
			*Lecane inermis*			*Lecane*	*Lecane luna*
			*Lecane ungulata*			*Lecane*	*Lecane inermis*
		*Monostyla*	*Monostyla* sp. ^1^			*Lecane*	*Lecane niothis*
	Notommatidae	*Cephalodella*	*Cephalodella forficula*			*Lecane*	*Lecane* sp.^1^
		*Monommata*	*Monommata maculata*			*Lecane*	*Lecane ungulata*
			*Notommata allantois*		Notommatidae	*Cephalodella*	*Cephalodella gibba*
			*Notommata codonella*			*Eothinia*	*Eothinia elongata*
	Philodinidae	*Anomopus*	*Anomopus telphusae*			*Notommata*	*Notommata tripus*
		*Philodina*	*Philodina megalotrocha*			*Rhinoglena*	*Rhinoglena frontalis*
		*Rotaria*	*Rotaria rotatoria*			*Scaridium*	*Scaridium longicauda*
	Proalidae	*Proales*	*Proales doliaris*		Philodinidae	*Rotaria*	*Rotaria neptunia*
	Synchaetidae	*Macrochaetus*	*Macrochaetus collinsii*			*Rotaria*	*Rotaria rotatoria*
		*Ploesoma*	*Ploesoma hudsoni*		Synchaetidae	*Ploesoma*	*Ploesoma hudsoni*
			*Ploesoma truncatum*			*Ploesoma*	*Ploesoma truncatum*
		*Polyarthra*	*Polyarthra dolichoptera*			*Polyarthra*	*Polyarthra dolichoptera*
			*Polyarthra remata*			*Polyarthra*	*Polyarthra euryptera*
		*Synchaeta*	*Synchaeta pectinata*			*Polyarthra*	*Polyarthra trigla*
			*Synchaeta tremula*			*Polyarthra*	*Polyarthra vulgaris*
	Scaridiidae	*Scaridium*	*Scaridium longicauda*			*Synchaeta*	*Synchaeta grandis*
	Testudinellidae	*Hexarthra*	*Hexarthra intermedia*			*Synchaeta*	*Synchaeta longipes*
			*Hexarthra mira*			*Synchaeta*	*Synchaeta oblonga*
		*Testudinella*	*Testudinella patina*			*Synchaeta*	*Synchaeta pectinata*
			*Testudinella* sp. ^1^			*Synchaeta*	*Synchaeta tremula*
	Trichocercidae	*Trichocerca*	*Trichocerca elongata*		Testudinellidae	*Filinia*	*Filinia longiseta*
			*Trichocerca rattus*			*Filinia*	*Filinia passa*
			*Trichocerca tenuior*			*Hexarthra*	*Hexarthra mira*
	Trichotriidae	*Trichotria*	*Trichotria tetractis*		Trichocercidae	*Trichocerca*	*Trichocerca capucina*
Copepods	-	*-*	*Cyclops* sp. ^1^			*Trichocerca*	*Trichocerca cylindrica*
	Acantholeberis	*Acantholeberis*	*Acantholeberis curvirostris*			*Trichocerca*	*Trichocerca longiseta*
	Canthocamptidae	*Attheyella*	*Attheyella crassa*			*Trichocerca*	*Trichocerca lophoessa*
	Centropagidae	*Sinocalanus*	*Sinocalanus sinensis*			*Trichocerca*	*Trichocerca rattus*
			*Sinocalanus tenellus*			*Trichocerca*	*Trichocerca similis*
		*Limnocalanus*	*Limnocalanus macrurus*			*Trichocerca*	*Trichocerca weberi*
	Cyclopidae	*Acanthocyclops*	*Acanthocyclops bicuspidatus*			*Trichotria*	*Trichotria pocillum*
			*Acanthocyclops galbinus*			*Trichotria*	*Trichotria tetractis*
		*Diacyclops*	*Diacyclops jasnitskii*	Copepods	-	*-*	Copepod nauplii
			*Diacyclops* sp. ^1^		Centropagidae	*Sinocalanus*	*Sinocalanus dorrii*
		*Ectocyclops*	*Ectocyclops polyspinosus*		Cyclopidae	*Cyclopidae*	*Cyclopidae vicinus*
		*Eucyclops*	*Eucyclops dumonti*			*Eucyclops*	*Eucyclops speratus*
			*Eucyclops serrulatus*		Cletodidae	*Limnocletodes*	*Limnocletodes behningi*
			*Eucyclops speratus*		Cyclopidae	*Mesocyclops*	*Mesocyclops leuckarti*
			*Eucyclops macruroides*			*Microcyclops*	*Microcyclops varicans*
			*Eucyclops* sp. ^1^			*Thermocyclops*	*Thermocyclops taihokuensis*
		*Megacyclops*	*Megacyclops viridis*		Diaptomidae	*Neodiaptomus*	*Neodiaptomus schmackeri*
		*Mesocyclops*	*Mesocyclops dissimilis*			*Neutrodiaptomus*	*Neutrodiaptomus incongruens*
			*Mesocyclops leuckarti*		Pseudodiaptomidae	*Schmackeria*	*Schmackeria forbesi*
			*Mesocyclops pehpeiensis*	Cladocerans	Bosminidae	*Bosmina*	*Bosmina coregoni*
		*Microcyclops*	*Microcyclops varicans*			*Bosmina*	*Bosmina longirostris*
		*Neodiaptomus*	*Neodiaptomus schmackeri*			*Bosminopsis*	*Bosminopsis deitersi*
		*Thermocyclops*	*Thermocyclops* sp. ^1^		Chydoridae	*Alona*	*Alona guttata*
			*Thermocyclops crassus*			*Alona*	*Alona rectangula*
			*Thermocyclops decipiens*			*Chydorus*	*Chydorus sphaericus*
		*Tropocyclops*	*Tropocyclops ishidai*		Daphniidae	*Ceriodaphnia*	*Ceriodaphnia quadrangula*
		*Paracyclops*	*Paracyclops fimbriatus*			*Diaphnia*	*Diaphnia cucullata*
	Diaptomidae	*Acanthodiaptomus*	*Acanthodiaptomus pacificus*		Leptodoridae	*Leptodora*	*Leptodora kindtii*
		*Arctodiaptomus*	*Arctodiaptomus stephanidesi*		Macrothricidae	*Ilyocryptus*	*Ilyocryptus sordidus*
			*Arctodiaptomus wierzejskii*		Moinidae	*Moina*	*Moina micrura*
		*Paracyclopina*	*Paracyclopina nana*		Sididae	*Diaphanosoma*	*Diaphanosoma brachyurum*
		*Sinodiaptomus*	*Sinodiaptomus sarsi*			*Diaphanosoma*	*Diaphanosoma leuchtenbergianum*
	Ergasilidae	*Ergasilus*	*Ergasilus hypomesi*				
		*Neoergasilus*	*Neoergasilus japonicus*				
		*Pseudergasilus*	*Pseudergasilus parasiluri*				
		*Sinergasilus*	*Sinergasilus polycolpus*				
	Lernaeidae	*Lernaea*	*Lernaea cyprinacea*				
	Pseudodiaptomidae	*Pseudodiaptomus*	*Pseudodiaptomus inopinus*				
Cladocerans	Chydoridae	*Chydorus*	*Chydorus sphaericus*				
	Sididae	*Diaphanosoma*	*Diaphanosoma* sp. ^1^				

−: the genus and family names are unknown. Sp.^1^: a species of the genus or class.

**Table A2:** The Family of MBC and MOI and their abbreviations.

Family of MBC	Abbreviation	Family of MOI	Abbreviation
Acantholeberis	ACAN	Asplanchnidae	ASPL
Asplanchnidae	ASPL	Brachionidae	BRAC
Brachionidae	BRAC	Bosmindae	BOSM
Canthocamptidae	CANT	Centropagidae	CENT
Centropagidae	CENT	Chydoridae	CHYD
Chydoridae	CHYD	Cletodidae	CLET
Collothecidae	COLL	Conochilidae	CONO
Conochilidae	CONO	Cyclopidae	CYCL
Cyclopidae	CYCO	Daphniidae	DAPH
Diaptomidae	DIAP	Dicranophoridae	DICR
Dicranophoridae	DICR	Diaptomidae	DIAP
Ergasilidae	ERGA	Gastropodidae	GAST
Flosculariidae	FLOS	Lecanidae	LECA
Gastropidae	GAST	Leptodoridae	LEPT
Lecanidae	LECA	Macrothricidae	MACR
Lernaeidae	LERN	Moindae	MOIN
Notommatidae	NOTO	Notommatidae	NOTO
Philodinidae	PHIL	Philodinidea	PHIL
Proalidae	PROA	Pseudodiaptomidae	PSEU
Pseudodiaptomidae	PSEU	Sididae	SIDI
Scaridiidae	SCAR	Synchaetidae	SYNC
Sididae	SIDI	Testudinellidae	TEST
Synchaetidae	SYNC	Trichocercidae	TRIC
Testudinellidae	TEST		
Trichocercidae	TRIC		
Trichotriidae	TRIH		

**Table A3:** The seasonal environment factors.

	WD	V	Turb	T	Sal	DO	Chl a	pH	TN	TP
SP	1.28 ± 0.05	0.11 ± 0.00	13.00 ± 1.86	22.29 ± 0.1	0.03 ± 0.00	10.21 ± 0.13	17.93 ± 0.65	7.10 ± 0.08	1.23 ± 0.06	0.13 ± 0.01
SU	3.38 ± 0.08	0.17 ± 0.01	13.84 ± 1.46	30.03 ± 0.13	0.11 ± 0.00	8.4 ± 0.09	16.32 ± 0.31	7.28 ± 0.01	1.22 ± 0.02	0.2 ± 0.01
A	1.46 ± 0.04	0.1 ± 0.00	49.46 ± 3.52	16.6 ± 0.26	0.02 ± 0.00	9.53 ± 0.16	19.68 ± 0.61	7.46 ± 0.06	1.28 ± 0.07	0.20 ± 0.01
W	0.86 ± 0.04	0.1 ± 0.00	129.92 ± 8.31	8.81 ± 0.13	0.03 ± 0.00	8.07 ± 0.09	16.54 ± 0.89	6.69 ± 0.04	3.63 ± 0.15	0.33 ± 0.03

## References

[1] Nie X., Hu X.R., Liu G., Jin B., Qin H. (2018). Species diversity of zooplankton and water quality biological assessment in a sub-lake of Poyang Lake. J. Nanchang Univ. Nat. Sci..

[2] Chen B., Chen L., Huang B., Michishita R., Xu B. (2021). Surface groundwater interaction in the dish lake wetland system of poyang Lake floodplain. J. Lake Sci..

[3] Lu Q., Hu X., Nie X., Ouyang S., Wang C., Qin H. (2020). Impact of water level fluctuations on the succession of zooplankton in Poyang lake. Acta Ecol. Sin..

[4] Tan C., Sheng T., Wang L., Mbao E., Gao J., Wang B. (2021). Water-level fluctuations affect the alpha and beta diversity of macroinvertebrates in Poyang Lake, China. Fundam. Appl. Limnol..

[5] Hu B., Hu X., Nie X., Zhang X., Wu N., Hong Y., Qin H.M. (2019). Seasonal and inter-annual community structure characteristics of zooplankton driven by water environment factors in a sub-lake of Lake Poyang, China. PeerJ.

[6] Gomes L.F., Pereira H.R., Gomes A.C.A.M., Vieira M.C., Martins P.R., Roitman I., Vieira L.C.G. (2019). Zooplankton functional-approach studies in continental aquatic environments: A systematic review. Aquat. Ecol..

[7] Mehner T., Keeling C., Emmrich M., Holmgren K., Argillier C., Volta P., Winfield I.J., Brucet S. (2016). Effects of fish predation on density and size spectra of prey fish communities in lakes. Can. J. Fish. Aquat. Science.

[8] Park K.S., Shin H.W. (2007). Studies on phyto-and-zooplankton composition and its relation to fish productivity in a west coast fish pond ecosystem. J. Environ. Biol..

[9] Korponai J., Braun M., Forró L., Gyulai I., Kövér C., Nédli J., Urák I., Buczkó K. (2019). Taxonomic, functional and phylogenetic diversity: How subfossil cladocerans mirror contemporary community for ecosystem functioning: A comparative study in two oxbows. Limnetica.

[10] Jeppesen E., Nõges P., Davidson T., Haberman J., Nõges T., Blank K., Lauridsen T., Søndergaard M., Sayer C., Laugaste R. (2011). Zooplankton as indicators in lakes: A scientific-based plea for including zooplankton in the ecological quality assessment of lakes according to the European Water Framework Directive (WFD). Hydrobiologia.

[11] Chen G., Dalton C., Taylor D. (2010). Cladocera as indicators of trophic state in Irish lakes. J. Paleolimnol..

[12] Ekvall M.K., Urrutia-Cordero P., Hansson L.-A. (2014). Linking Cascading Effects of Fish Predation and Zooplankton Grazing to Reduced Cyanobacterial Biomass and Toxin Levels Following Biomanipulation. PLoS ONE.

[13] Berta C., Tóthmérész B., Wojewódka M., Augustyniuk O., Korponai J., Bertalan-Balázs B., Nagy A.S., Grigorszky I., Gyulai I., Balázs B. (2019). Community Response of Cladocera to Trophic Stress by Biomanipulation in a Shallow Oxbow Lake. Water.

[14] Montes-Ortiz L., Elias-Gutierrez M. (2018). Faunistic survey of the zooplankton community in an oligotrophic sinkhole, Cenote Azul (Quintana Roo, Mexico), using different sampling methods, and documented with DNA barcodes. J. Limnol..

[15] Choquet M., Kosobokova K., Kwaśniewski S., Hatlebakk M., Dhanasiri A.K.S., Melle W., Daase M., Svensen C., Søreide J.E., Hoarau G. (2018). Can morphology reliably distinguish between the copepods *Calanus finmarchicus* and *C. glacialis*, or is DNA the only way?. Limnol. Oceanogr. Methods.

[16] Abad D., Albaina A., Aguirre M., Laza-Martínez A., Uriarte I., Iriarte A., Villate F., Estonba A. (2016). Is metabarcoding suitable for estuarine plankton monitoring? A comparative study with microscopy. Mar. Biol..

[17] Van der Loos L.M., Nijland R. (2021). Biases in bulk: DNA metabarcoding of marine communities and the methodology involved. Mol. Ecol..

[18] Stefanni S., Stanković D., Borme D., De Olazabal A., Juretić T., Pallavicini A., Tirelli V. (2018). Multi-marker metabarcoding approach to study mesozooplankton at basin scale. Sci. Rep..

[19] Chain F.J., Brown E.A., MacIsaac H.J., Cristescu M.E. (2016). Metabarcoding reveals strong spatial structure and temporal turnover of zooplankton communities among marine and freshwater ports. Divers. Distrib..

[20] Deagle B.E., Jarman S.N., Coissac E., Pompanon F., Taberlet P. (2014). DNA metabarcoding and the cytochrome c oxidase subunit I marker: Not a perfect match. Biol. Lett..

[21] Zhang X.W. (2019). Environmental DNA Shaping a New Era of Ecotoxicological Research. Environ. Sci. Technol..

[22] Yang J., Zhang X., Xie Y., Song C., Zhang Y., Yu H., Burton G.A. (2017). Zooplankton Community Profiling in a Eutrophic Freshwater Ecosystem-Lake Tai Basin by DNA Metabarcoding. Sci. Rep..

[23] Yang J., Zhang X., Zhang W., Sun J., Xie Y., Zhang Y., Burton G.A., Yu H. (2017). Indigenous species barcode database improves the identification of zooplankton. PLoS ONE.

[24] Bucklin A., Lindeque P.K., Rodriguez-Ezpeleta N., Albaina A., Lehtiniemi M. (2016). Metabarcoding of marine zooplankton: Prospects, progress and pitfalls. J. Plankton Res..

[25] Casas L., Pearman J.K., Irigoien X. (2017). Metabarcoding Reveals Seasonal and Temperature-Dependent Succession of Zooplankton Communities in the Red Sea. Front. Mar. Sci..

[26] Zhang G.K., Chain F.J.J., Abbott C.L., Cristescu M.E. (2018). Metabarcoding using multiplexed markers increases species detection in complex zooplankton communities. Evol. Appl..

[27] Chaparro G., O’Farrell I., Hein T. (2019). Multi-scale analysis of functional plankton diversity in floodplain wetlands: Effects of river regulation. Sci. Total Environ..

[28] Pennak R.W. (1989). Freshwater Invertebrates of the United States.

[29] Witty L.M. (2004). Practical Guide to Identifying Freshwater Crustacean Zooplankton.

[30] Shiel R.J. (1995). A Guide to Identification of Rotifers, Cladocerans and Copepods from Australian Inland Waters.

[31] Phan D.D., Nguyen V.K., Nga N., Thi L., Ngoc T.D., Hai H.T. (2015). Identification Handbook of Freshwater Zooplankton of the Mekong River and its Tributaries.

[32] Wang J.J. (1961). Fauna Sinica: Freshwater Rotifera.

[33] Jiang Y.Z., Du N.S. (1979). Fauna Sinica (Crustacea): Freshwater Cladocera.

[34] Zhang Z.S., Huang X.F. (1995). Research Methods of Freshwater Plankton.

[35] Institute of Zoology, Chinese Academy of Sciences (1979). Fauna Sinica (Crustacea): Freshwater Copepods.

[36] Han M.S., Shu W.F. (1995). Picture of Chinese Freshwater Biota.

[37] Pilliod D.S., Goldberg C.S., Arkle R.S., Waits L.P. (2013). Estimating occupancy and abundance of stream amphibians using environmental DNA from filtered water samples. Can. J. Fish. Aquat. Sci..

[38] Xiong W., Ni P., Chen Y., Gao Y., Shan B., Zhan A. (2017). Zooplankton community structure along a pollution gradient at fine geographical scales in river ecosystems: The importance of species sorting over dispersal. Mol. Ecol..

[39] Borcard D., Gillet F., Legendre L. (2011). Numerical Ecology with R.

[40] Novotny A., Zamora-Terol S., Winder M. (2021). DNA metabarcoding reveals trophic niche diversity of micro and mesozooplankton species. Proc. R. Soc. B.

[41] Ren Z., Qu X., Zhang M., Yu Y., Peng W. (2019). Distinct Bacterial Communities in Wet and Dry Seasons During a Seasonal Water Level Fluctuation in the Largest Freshwater Lake (Poyang Lake) in China. Front. Microbiol..

[42] Barnett A., Beisner B. (2007). zooplankton biodiversity and lake trophic state: Explanations invoking resource abundance and distribution. Ecology.

[43] Zhou J., Qin B., Zhu G., Zhang Y., Gao G. (2020). Long-term variation of zooplankton communities in a large, heterogenous lake: Implications for future environmental change scenarios. Environ. Res..

[44] Wang S., Gao Y., Jia J., Kun S., Lyu S., Li Z., Lu Y., Wen X. (2021). Water level as the key controlling regulator associated with nutrient and gross primary productivity changes in a large floodplain-lake system (Lake Poyang), China. J. Hydrol..

[45] Qian K., Dokulil M., Lei W., Chen Y. (2021). The effects of water-level changes on periphytic algal assemblages in Poyang Lake. Fundam. Appl. Limnol..

[46] Liu B., Liu J., Jeppesen E., Chen Y., Liu X., Zhang W. (2019). Horizontal distribution of pelagic crustacean zooplankton biomass and body size in contrasting habitat types in Lake Poyang, China. Environ. Sci. Pollut. Res..

[47] Cao J., Chu Z., Du Y., Hou Z., Wang S. (2016). Phytoplankton dynamics and their relationship with environmental variables of Lake Poyang. Hydrol. Res..

[48] Zamora-Terol S., Novotny A., Winder M. (2020). Reconstructing marine plankton food web interactions using DNA metabarcoding. Mol. Ecol..

[49] Amorim C.A., Moura A.N. (2020). Ecological impacts of freshwater algal blooms on water quality, plankton biodiversity, structure, an. ecosystem functioning. Sci. Total Environ..

[50] Gołdyn R., Kowalczewska-Madura K. (2008). Interactions between phytoplankton and zooplankton in the hypertrophic Swarzedzkie Lake in western Poland. J. Plankton Res..

[51] Špoljar M., Dražina T., Šargač J., Borojević K.K., Žutinić P. (2012). Submerged macrophytes as a habitat for zooplankton development in two reservoirs of a flow-through system (Papuk Nature Park, Croatia). Ann. Limnol. Int. J. Lim..

[52] Sharma P., Gutierrez M.E., Kobayashi T. (2014). Identification of common cladocerans and calanoids in two south Australian reservoirs using DNA barcoding and morphological analysis: An integrative approach. Crustaceana.

[53] Schroeder A., Stanković D., Pallavicini A., Gionechetti F., Pansera M., Camatti E. (2020). DNA metabarcoding and morphological analysis—Assessment of zooplankton biodiversity in transitional waters. Mar. Environ. Res..

[54] Walczyńska K.S., Søreide J.E., Weydmann-Zwolicka A., Ronowicz M., Gabrielsen T.M. (2019). DNA barcoding of Cirripedia larvae reveals new knowledge on their biology in Arctic coastal ecosystems. Hydrobiologia.

[55] Frontalini F., Cordier T., Balassi E., du Chatelet E.A., Cermakova K., Apothéloz-Perret-Gentil L., Martins M.V.A., Bucci C., Scantamburlo E., Treglia M. (2020). Benthic foraminiferal metabarcoding and morphology-based assessment around three offshore gas platforms: Congruence and complementarity. Environ. Int..

[56] Elías-Gutiérrez M., Valdez-Moreno M., Topan J., Young M.R., Cohuo-Colli J.A. (2018). Improved protocols to accelerate the assembly of DNA barcode reference libraries for freshwater zooplankton. Ecol. Evol..

[57] Cordier T., Lanzén A., Apothéloz-Perret-Gentil L., Stoeck T., Pawlowski J. (2019). Embracing Environmental Genomics and Machine Learning for Routine Biomonitoring. Trends Microbiol..

[58] Banerji A., Bagley M., Elk M., Pilgrim E., Martinson J., Domingo J.S. (2018). Spatial and temporal dynamics of a freshwater eukaryotic plankton community revealed via 18S rRNA gene metabarcoding. Hydrobiologia.

[59] Djurhuus A., Pitz K., Sawaya N., Rojas-Márquez J., Michaud B., Montes E., Muller-Karger F., Breitbart M. (2018). Evaluation of marine zooplankton community structure through environmental DNA metabarcoding. Limnol. Oceanogr. Methods.

[60] Makino W., Maruoka N., Nakagawa M., Takamura N. (2017). DNA barcoding of freshwater zooplankton in Lake Kasumigaura, Japan. Ecol. Res..

[61] Shackleton M., Dafforn K., Murphy N., Greenfield P., Cassidy M., Besley C. (2021). How does molecular taxonomy for deriving river health indices correlate with traditional morphological taxonomy?. Ecol. Indic..

[62] Lim N.K.M., Tay Y.C., Srivathsan A., Tan J.W.T., Kwik J.T.B., Baloglu B., Meier R., Yeo D.C.J. (2016). Next-generation freshwater bioassessment: eDNA metabarcoding with a conserved metazoan primer reveals species-rich and reservoir-specific communities. R. Soc. Open Sci..

[63] Nistal-García A., García-García P., García-Girón J., Borrego-Ramos M., Blanco S., Bécares E. (2021). DNA metabarcoding and morphological methods show complementary patterns in the metacommunity organization of lentic epiphytic diatoms. Sci. Total Environ..

[64] Leray M., Knowlton N., Ho S.-L., Nguyen B.N., Machida R.J. (2019). GenBank is a reliable resource for 21st century biodiversity research. Proc. Natl. Acad. Sci. USA.

